# Inequities in child protective services contact among First Nations and non-First Nations parents in one Canadian province: a retrospective population-based study

**DOI:** 10.1186/s12889-025-21813-5

**Published:** 2025-04-02

**Authors:** Kathleen S. Kenny, Elizabeth Wall-Wieler, Kayla Frank, Lindey Courchene, Mary Burton, Michael Champagne, Marlyn Bennett, Cathy Rocke, Marni Brownell, Marcelo L. Urquia

**Affiliations:** 1 Manitoba Centre for Health Policy, University of Manitoba, Winnipeg, Canada; 2 Department of Community Health Sciences, Rady Faculty of Health Sciences, University of Manitoba, Winnipeg, Canada; 3 First Nations Family Advocate Office, Winnipeg, Canada; 4 Zoongizi Ode, Winnipeg, Canada; 5 Faculty of Social Work, University of Calgary, Calgary, Canada; 6 Faculty of Social Work, University of Regina, Regina, Canada

**Keywords:** First Nations, Indigenous, Child protective services, Child welfare, Parents, Mothers, Child removal, Surveillance, Racism, Colonialism

## Abstract

**Background:**

Parental contact with child protective services (CPS) has been linked to deteriorating health among parents. Capturing rates of CPS contact among parents is therefore important for understanding inequities in exposure and their potential role in amplifying racial inequities in health and wellbeing. Though an extensive body of research in North America has provided population-level analyses of CPS contact among children, a disproportionate percentage of whom are Indigenous, no studies to date have extrapolated estimates to account for contact in parent populations, leading to a fragmented view of the system’s reach and impact beyond the child-level. In order to advance health equity-oriented research in this domain, our study calculated previously unexplored population-level estimates of CPS contact among First Nations and non-First Nations parents.

**Methods:**

We used whole-population linked data from Manitoba (Canada) to identify 119,883 birthing parents (13,171 First Nations; 106,712 non-First Nations) who had their first child between 1998 and 2019. We calculated prevalence rates, rate differences, and rate ratios of parental contact with different levels of CPS by First Nations status (categorization used in Canada for Indigenous peoples who are members of a First Nation), including ever had an open CPS file for child(ren), ever had out-of-home placement of child(ren), and ever had termination of parental rights (TPR).

**Results:**

Overall, 49.6% of First Nations parents had a CPS file open for their child(ren) (vs. 13.1% among non-First Nations parents), 27.4% had out-of-home placement of their child(ren) (vs. 4.7% among non-First Nations parents), and 9.6% experienced TPR (vs. 1.8% among non-First Nations parents).

**Conclusions:**

CPS contact was high among parents and prevalence was almost 4 times higher among First Nations parents, where 1 out of 2 were intervened upon by CPS. Findings reinforce significant concerns about the system’s scope and the crucial importance of considering its role in compounding health inequities and sustaining colonialism in Canada. First Nations-led interventions are needed to reduce CPS disruption to the lives of First Nations peoples.

**Supplementary Information:**

The online version contains supplementary material available at 10.1186/s12889-025-21813-5.

## Background

North America’s child protective services’ (CPS) disproportionately intervene in the lives of Indigenous and Black families; a phenomenon that is a well-recognized manifestation of colonialism and systemic racism [1–6]. As a unique state structure with dual responsibilities of child protection and social control, CPS interventions exist along a continuum from home monitoring to family support services and referrals, to at its most severe, child removal and termination of parental rights. Although an extensive body of demographic research has provided analyses of population-level CPS contact among children [7–10], including by race/ethnicity [1, 11, 12], no studies to date have extrapolated estimates to account for contact among parents as shares of the population. Inattention to parental-level contact, including the extent of racial and ethnic inequities at different levels of CPS, has contributed to a fragmented view of the system’s reach and costs beyond child populations, including neglect of its potential impacts on parents’ health.

Among children, the most recent national data from Canada on the incidence of CPS events estimated that 4.8% of children 0–15 years old (or 300,000 children) experienced a CPS investigations, 1.6% were screened-in or had a substantiated investigation, and 0.3% experienced out-of-home placement or foster care in 2018 [13]. While comprehensive data on the race/ethnicities of children coming into contact with CPS in Canada are generally unavailable at the national-level, data from the same study on children with First Nations status (categorization used in Canada to describe Indigenous peoples who are members of a First Nation), found that First Nations children were 3.6 times more likely to experience an investigation, 4.7 times more likely to experience a substantiation, and 17.2 times more likely to experience an out-of-placement than non-Indigenous children [2]. Looking beyond incidence of CPS involvement to cumulative rates of CPS involvement, only a few studies in Canada have attempted to provide a wider view of this phenomenon across childhood [7, 14, 15], including one that examined rates among First Nations children in Manitoba, which reported that 22% of all First Nations children in the province experienced being removed from their homes by CPS before age 16, compared with 2% of non-First Nations children [15]. While together these data show CPS contact to be widespread with concerning inequities between First Nations and non-First Nations populations, their unique focus on quantifying child-level contact has precluded a fuller understanding of the system’s footprint, which is inherently multifaceted and thus more accurately accounted for by assessing its multi-level impacts on families and communities, including attention to CPS exposure among parents. For parents, CPS encounters are often described as significant events in individuals’ lives and have been associated with a host of severe challenges, including mental distress and trauma [16], stigma [17, 18], extreme fear of losing child custody [19, 20], stress related to system oversight and compliance [21], increased marginalization [22], and the potential of long-term sanctions by the system [25]. In cases of child removal, studies have also documented a range of harms to parents’ health, including increased suicidality, depression, anxiety, substance use [26, 27], and premature mortality [28, 29]. For many impacted parents, who are already under-resourced and often subject to structural disadvantages and systemic racism, CPS encounters can thus contribute to substantial hardships and widening inequities that may be distinctly jeopardizing for First Nations parents due to conditions of historical and ongoing colonialism in Canada [30, 31]. In light of these realities, a better understanding of the distribution and severity of CPS interventions among parents is important to provide a more accurate picture of the system’s scope and help identify opportunities for preventative and supportive community-based services [32, 33]. Addressing these gaps in understanding is also relevant to First Nations leadership, advocates, governments, impacted communities, scholars, and others who are considering the legacy and efficacy of CPS interventions, as well as remedies for mitigating their long term impacts on First Nations families and communities in Canada.

This paper addresses some of these gaps in the jurisdiction of Manitoba, a central Canadian province with approximately 1.4 million residents [34]. Manitoba is a key setting for studying levels of parental involvement with CPS because it is one of the only jurisdictions globally where linkable administrative data collected at the individual-level for all residents is linked to CPS databases, make it possible to reliably estimate parental contact with CPS at the whole-population level. Manitoba is also the Canadian province with the highest proportion of Indigenous peoples and has a rate of children in foster care that is over four-times the national average, with Indigenous families, particularly First Nations families severely over-represented [15, 35]. Notably, in the early 2000s, Manitoba was also the first province in Canada to recognize Indigenous peoples’ responsibility and authority to care for their children. This assertion was followed by new legislation in 2003 that transferred responsibility for the delivery of child and family services, including child protective services from a central authority to four regional authorities, including three Indigenous-controlled authorities [36]. While the goals of this de-centralization, which has continued to the present time, were to increase First Nations and Indigenous-led services and reduce the system’s over-representation of Indigenous children, the enforcement of colonial child protection legislation, the absence of legal mechanisms to assert traditional Indigenous laws and values, and funding shortfalls have significantly undermined First Nations sovereignty over child and family matters [37], resulting in the First Nations Chiefs passing a resolution through the Assembly of Manitoba Chiefs to withdraw from the implementation process in 2010 [38]. 

Importantly, like in other settler-colonial jurisdictions in Canada, contemporary CPS policies in Manitoba are also integrally connected to a much longer history of discriminatory oversight and forced family separation that has operated as a central modality of white supremacy and genocide of Indigenous peoples in North America since the 16th century [3, 39]. In this context, child removal, first instituted in the 1600s with the establishment of the Residential and Boarding School Systems in North America, and later through involuntary adoptions, have been and continue to be instrumental in upholding other forms of settler-colonial violence targeting Indigenous peoples’ assertions of sovereignty over self-governance, economic development, and land [40]. These cross-generational harms and explicitly racist systems of economic marginalization are also at the root of many of the structural inequities and challenges faced by First Nations and other Indigenous families in North America today [2].

Our study objective was to calculate previously unexplored population-level prevalence estimates of CPS contact among First Nations and non-First Nations parents. To do this, we drew on 20 years of data on all birthing people in Manitoba who had their first children between 1998 and 2019 and provide the prevalence rates of having a CPS file open, experiencing out-of-home placement of a child, and having parental rights terminated.

## Data and methods

### Study population

This study is part of a broader community-engaged research project with the overall objective of understanding the health needs of families involved with Child Protective Services in Manitoba, with a main focus on the health experiences of parents. This study and the broader project were developed in consultation with First Nations government, organizations serving First Nations and other Indigenous and non-Indigenous families, clinical and policy experts, and CPS-impacted parents and grandparents. It is guided by three advisory boards composed of: (1) First Nations government representatives; (2) First Nations and non-First Nations community organizations; and (3) CPS authorities.

### Data sources

Our study used linked administrative data in the Population Research Data Repository housed at the Manitoba Centre for Health Policy (MCHP). For this study, the Manitoba Health Insurance Plan registry was linked at the individual-level with case reports from the Child and Family Services Information System (CFSIS), hospital birth records, employment and income assistance case reports, and the Canadian Census. The registry includes information on all Manitobans registered for healthcare insurance (representing > 99% of Manitobans). A scrambled personal health number was used to link these de-identified datasets. Linkages between children and parents were identified through the Family Registration Number. Additional information on linkage methods, confidentiality/privacy, and validity are published elsewhere [41]. We used the First Nations Research File to identify First Nations in Manitoba (Anishinaabe, Nehethowuk, Denesuline, Anishininew, and Dakota Oyate) who are registered as Status Indians under the Federal Indian Act (1985) that was generated from the federal government Indian Status Registry as of 2016 and provided to MCHP by the First Nations Health and Social Secretariat of Manitoba (FNHSSM). Our study received ethical approval from the University of Manitoba’s Health Research Ethics Board (HS24504 H2020-528) and the Health Information Research Governance Committee of FNHSSM (approved April 2021), and privacy and confidentiality approval from the Government of Manitoba’s Health Information Privacy Committee (2020/2021-65).

### Cohorts

This study included the entire population of birthing people who had their first child in Manitoba during the period of April 1, 1998, to March 31, 2019. Of the 119,883 parents identified, 13,171 (11%) were identified as First Nations through the Manitoba First Nations Research File. A sub-cohort of birthing parents was also identified and included those who had all their children between April 1, 1998, and March 31, 2009, and who lived in Manitoba until at least March 31, 2019. In this cohort of 1,911 birthing persons who were First Nations and 30,663 who were non-First Nations, all children were at least 10 years old at the end of the study period, making it a more complete representation of parental contact with the system.

### Levels of contact with CPS

#### Ever had a CPS file open for child(ren)

Using CPS case reports, we ascertained whether a parent “ever had a CPS file open for child(ren)” based on: (a) a CPS file being open on a birthing parent any time at or after 9 months before the birth of their first child; or (b) a CPS file being open for any of the birthing person’s biological children after their birth; or (c) a CPS placement record for any of the birthing person’s biological children after their birth.

#### Ever had child(ren) in out-of-home placement

We defined “ever had child(ren) in out-of-home placement” as the first event of out-of-home placement of at least one biological child. This information was obtained from CFSIS case reports, with an episode of placement assessed based on placement start date and end date.

#### Termination of Parental Rights (TPR)

Termination of parental rights (TPR) was defined as the first event of legal termination of parental rights based on information from CFSIS about the child’s legal status as ‘permanent ward’. This outcome marks the severing of the legal bond between a parent and child, and the likely end of attempts for legal reunification, though in some cases it may be reversed.

#### Other descriptive characteristics

We also examined several other characteristics of parents at birth of first child, including age, neighborhood location (urban, rural), neighborhood income quintile, as well as the off-on reservation status of First Nations parents, and age at first parental contact with CPS.

### Statistical analyses

Using all First Nations and non-First Nations birthing persons as denominators, we calculated the period prevalence of parents’ exposure to each level of CPS contact, and compared the distribution of parent characteristics according to level of contact. In order to quantify relative and absolute population-level inequities in CPS involvement between First Nations and non-First Nations parents we calculated rate ratios (RR) and rate differences (RD) and corresponding 95% confidence interval (CI) using the Cochran-Mantel-Haenszel approach.Theseunadjusted measures aim to capture the direct exposure of parents to the CPS system.

As a sensitivity analysis we calculated the prevalence rates of CPS contact in a sub-cohort of First Nations and non-First Nations parents who had all their children between April 1, 1998, and March 31, 2009, and who lived in Manitoba until at least March 31, 2019. This extended period of follow-up allowed us to assess to what extent our main analysis may have underestimated the number of parents experiencing CPS contact.

All data management and analyses were performed using SAS (version 9.4; SAS Institute, Cary, NC).

## Results

Of the 13,171 First Nations parents, 6531 (49.6%) had a CPS file open for their child(ren), 3609 (27.4%) had out-of-home placement of their child(ren), and 1259 (9.6%) experienced TPR. CPS involvement among First Nations parents was higher among parents who had their first child before age 20, who lived on a First Nations reservation, and who lived in rural and low-income neighborhoods (Table 1). Of the 106,712 non-First Nations parents, 13,944 *(*13.1%) had a file open, 5029 (4.7%) had out-of-home placement of their child(ren), and 1963 (1.8%) experienced TPR. CPS involvement among these parents was highest for those who had their first child before age 20, and those who lived in urban and low-income neighborhoods (Table 1).

**Table 1:** Characteristics of First Nations and non-First Nations birthing persons by level of parental contact with CPS, 1998–2019

	First Nations birthing persons					Non-First Nations birthing persons			
	*N* = 13,171					*N* = 106,712			
	No CPS involvement	Ever had a CPS file open for child(ren)	Ever had child(ren) in out-of-home placement	Ever had termination of parental rights		No CPS involvement	Ever had a CPS file open for child(ren)	Ever had child(ren) in out-of-home placement	Ever had termination of parental rights
	n (%)	n (%)	n (%)	n (%)		n (%)	n (%)	n (%)	n (%)
**All n (% of N)**	6640 (50.4)	6531 (49.6)	3609 (27.4)	1259 (9.6)		92,768 (86.9)	13,944 (13.1)	5029 (4.7)	1963 (1.8)
Age at first parental involvement with CPS^^^									
Under 18		1514 (23.2)	812 (22.5)	359(28.5)			3043 (21.8)	1153 (22.9)	508 (25.9)
18–19		781 (12.0)	526 (14.6)	232 (18.4)			1424 (10.2)	764 (15.2)	392 (20.0)
20–29		3276 (50.2)	1880 (52.1)	598 (47.5)			5661 (40.6)	2281 (45.4)	882 (44.9)
30+		953 (14.6)	387 (10.7)	67 (5.3)			3741 (26.8)	791 (15.7)	181 (9.2)
**Characteristics at first child’s birth**									
Age^*^									
Under 18	1183 (17.8)	2481 (38.0)	1379 (38.2)	520 (41.3)		697 (0.8)	3428 (24.6)	1330 (26.5)	544 (27.7)
18–19	1853 (27.9)	1843 (28.2)	1064 (29.5)	377 (29.9)		4846 (5.2)	3033 (21.7)	1364 (27.1)	588 (30.0)
20–29	3245 (48.9)	2075 (31.8)	1106 (30.6)	342 (27.2)		53,967 (58.2)	6006 (43.1)	1984 (39.4)	727 (37.0)
30+	359 (5.4)	125 (1.9)	56 (1.6)	17 (1.4)		32,878 (35.4)	1402 (10.1)	311 (6.2)	84 (4.3)
Neighborhood Location									
Urban	713 (10.7)	1,212 (18.6)	686 (19.0)	319 (25.3)		60,704 (65.4)	8811 (63.2)	3093 (61.5)	1276 (65.0)
Rural	5898 (88.8)	5,272 (80.7)	2896 (80.2)	926 (73.6)		31,544 (34.0)	4899 (35.1)	1793 (35.7)	627 (31.9)
Missing	29 (0.5)	47 (0.7)	27 (0.8)	14 (1.1)		520 (0.6)	234 (1.7)	143 (2.8)	60 (3.1)
Reserve status									
Living on reserve	5195 (81.4)	4495 (72.3)	2491 (72.5)	779 (65.5)					
Living off reserve	1187 (18.6)	1719 (27.7)	943 (27.5)	410 (34.5)					
Neighborhood Income Quintile									
1 – Lowest	3,628 (54.7)	4,197 (64.3)	2331 (64.6)	836 (66.4)		16,283 (17.5)	4996 (35.8)	2212 (44.0)	964 (49.1)
2	1,899 (28.6)	1,345 (20.6)	703 (19.5)	237 (18.8)		19,589 (21.1)	3243 (23.3)	1101 (22.0)	419 (21.3)
3	380 (5.7)	422 (6.5)	269 (7.4)	110 (8.7)		19,811 (21.4)	2348 (16.8)	704 (14.0)	242 (12.3)
4	572 (8.6)	428 (6.5)	233 (6.5)	51 (4.1)		19,352 (20.9)	1844 (13.2)	521 (10.3)	162 (8.3)
5 – Highest	132 (2.0)	92 (1.4)	46 (1.3)	11 (0.9)		17,213 (18.5)	1279 (9.2)	348 (6.9)	116 (5.9)
Missing	29 (0.4)	47 (0.7)	27 (0.7)	14 (1.1)		520 (0.6)	234 (1.7)	143 (2.8)	60 (3.1)

^*n* = 82 are missing age at first parental contact with CPS

*n = 464 are missing age at first child’s birth

Comparing First Nations parents to other parents, First Nations parents were almost four-times (RR 3.80; 95% CI: 3.71, 3.88) times as likely to have a CPS file open, close to 6-times (RR 5.81; 95% CI: 5.59, 6.04) as likely to have out-of-home placement of their child(ren), and over 5-times (RR 5.20; 95% CI: 4.85, 5.56) as likely to experience TPR (Table 2). On the risk difference scale, the excess risk of having a CPS file open was 36.52% points (95% CI: 36.50, 36.54) higher for First Nations parents versus non-First Nations parents, 22.69% points (95% CI: 22.67, 22.70) higher for child removal, and 7.72% points higher for TPR (95% CI: 7.71, 7.73).

**Table 2:** Inequities in parental CPS contact among First Nations and non-First Nations birthing persons, 1998–2019

	Total *N* = 119,883	Level of CPS contact											
		Ever had a CPS File open for child(ren)				Ever had child(ren) in out-of-home placement				Ever had termination of parental rights			
		Yes *n* = 20,475	No *n* = 99,408	RD (95% CI)	RR (95% CI)	Yes *n* = 8638	No *n* = 111,245	RD (95% CI)	RR (95% CI)	Yes *n* = 3222	No *n* = 116,661	RD (95% CI)	RR (95% CI)
	n (% of N)	n (%)	n (%)			n (%)	n (%)			n (%)	n (%)		
First Nations birthing persons	13,171 (11.0)	6531 (31.9)	6640 (6.7)	36.52 (36.50, 36.54)	3.80 (3.71, 3.88)	3609 (41.8)	9562 (8.6)	22.69 (22.67, 22.70)	5.81 (5.59, 6.04)	1259 (39.1)	11,912 (10.2)	7.72 (7.71, 7.73	5.20 (4.85, 5.56)
Non-First Nations birthing persons*	106,712 (89.0)	13,944 (68.1)	92,768 (93.3)	1.00 (ref.)	1.00 (ref.)	5029 (58.2)	101,683 (91.4)	1.00 (ref.)	1.00 (ref.)	1963 (60.9)	104,749 (89.8)	1.00 (ref.)	1.00 (ref.)

*includes non-Status First Nations, Metis, Inuit, and non-Indigenous birthing persons

RD, rate difference

RR, rate ratio

CI, confidence interval

In a sensitivity analysis comparing results from our main analysis the sub-cohort of parents who had all their children between April 1, 1998, and March 31, 2009 (Table 3), prevalence rates from the sub-cohort were generally lower than the full cohort, suggesting that the main analysis is underestimating overall rates of system contact.

**Table 3:** Inequities in CPS contact in sub-cohort of First Nations and non-First Nations birthing persons who had all children between 1998 and 2010

	First Nations birthing persons					Non-First Nations birthing persons			
	*N* = 1,911					*N* = 30,663			
	No CPS involvement	Ever had a CPS file open for child(ren)	Ever had child(ren) in out-of-home placement	Ever had termination of parental rights		No CPS involvement	Ever had a CPS file open for child(ren)	Ever had child(ren) in out-of-home placement	Ever had termination of parental rights
	n (%)	n (%)	n (%)	n (%)		n (%)	n (%)	n (%)	n (%)
**All n (% of N)**	982 (51.4)	929 (48.6)	469 (24.4)	182 (9.5)		26,431 (86.9)	4,232(13.8)	1,156 (3.8)	424 (1.4)

## Discussion

Using 20 years of linked administrative data for all First Nations and non-First Nations birthing parents in one Canadian province, our analysis aimed to fill a gap in empirical research by estimating population-level prevalence of CPS contact among parents. We found that 50% of all First Nations parents had a CPS file open, 27% experienced child removal, and 10% experienced the termination of their parental rights compared with substantially lower CPS involvement among non-First Nations parents (13.1% had a file open; 4.7% experienced child removal; and 1.8% experienced TPR).

Our results provide further evidence that CPS interventions are widespread in the lives of parents and strikingly more common among First Nations parents. This significant disproportionality was most evident in the prevalence of having a CPS file open that was experienced by 1 in every 2 First Nations parents (compared to 1 in 8 non-First Nations parents). Though potentially a less severe intervention than later-stage sanctions like child removal or TPR, an open file (without other sanctions) often encompasses the provision of CPS services. These services, however, are frequently not deemed helpful by parents and coupled with the threat of child removal, involuntary surveillance, and hardships associated with navigating institutional processes - can often be experienced as straining. For parents, these experiences may also generate varying levels of psychological stress, trauma, and fear that can lead to disengagement from support services and reinforce the marginalization of already marginalized families [16, 22]. For First Nations families, this often coercive form of intervention is likely to be distinctly strenuous because of the documented intergenerational trauma and indignities that many First Nations families have cumulatively experienced through this system. These stresses also have the potential to be compounded by the geographic concentration of CPS interventions in First Nations communities, which are likely to intensify CPS surveillance and make system avoidance or exit more difficult or impossible. This spatial concentration of system involvement among First Nations peoples may also impose additional burdens at the community-level that are similar to the spill-over effects of mass incarceration, whereby mechanisms underlying collective well-being [42] and health [43] of families non-involved with CPS are also indirectly impacted [44].

Our findings showing rates of out-of-home placement and TPR to be over 5 times higher for First Nations parents further point to the large and severe impacts of this system on family life, and the pervasive challenge of systemic racism that disproportionately over-represents First Nations families at every level of this system. In absolute terms, our finding that child removal affected more than one quarter (27.4%) of the First Nations parent population sheds additional light on the mass injustice experienced by First Nations parents and their children by the hand of this system that has only previously been estimated at the child-level. Notably, the rates of child removal found in our study were higher than childhood estimates previously published from Manitoba [15], which though measuring a different period prevalence, suggest the population-level occurrence of CPS contact in a family is more accurately accounted for at the parent-level [45]. For parents, these events of family separation, described in prior research as one of the most traumatic and worst forms of institutional punishment [46] and detrimental to health [29, 47, 48], may also push already marginalized parents into more precarious positions, such as homelessness, that can be especially jeopardizing for First Nations and other Indigenous parents [23].

To understand the injustice of these outcomes for First Nations parents, it is crucial to also consider our findings as an extension of centuries of anti-Indigenous genocidal processes [3, 39] and oppressive system involvement of Indigenous families that have been instrumental in separating families and expanding other forms of colonial dispossession in Canada, including the theft of Indigenous lands [40]. These colonial forces, and the anti-Indigenous racism at their core, have also severely restricted the distribution of basic resources and funding for First Nations families and First Nations-led family service organizations – all of which have implications for the high levels of CPS contact and family separation among First Nations parents found in our study. At a local level, patterns of system contact may also be related, in part, to changes following the Manitoba government’s decentralization of CPS in 2003 that was characterized by shifts in CPS responsibilities to First Nations communities despite funding shortfalls and provincial legislative impediments to First Nations self-determination over child and family matters [37]. Another notable policy shift that may explicate our findings are the changes that were made to CPS workforce practices following public outrage over the system’s failures to prevent the 2005 murder of a 5 year-old First Nations child named Phoenix Sinclair, which resulted in revised organizational safety/risk assessments, including lower threshold decision-making relating to removing a child from their parent [49]. Paralleling these other local system changes, the high levels of CPS contact among First Nations parents are also likely attributable to a CPS-led prenatal reporting system in Canada, known as “Birth Alerts”. This “system” required healthcare providers at hospitals to notify CPS of an infant’s birth when CPS believed the newborn was at risk of harm or in need of protection. Though “Birth Alerts” ostensibly ended in Manitoba and many other parts of Canada in 2020, the “system”, which notably lacked formal policy and evidence-based practice guidelines, was in place for several decades, and was widely criticized by First Nations leadership to be discriminatory and to have resulted in the disproportionate removal First Nations and other Indigenous newborns from their parents. This “system” also placed parents at risk of CPS intervention in subsequent pregnancies and more broadly reinforced First Nations families’ mistrust of healthcare institutions [39].

Our study has implications for understanding the wide spectrum of ways in which interactions with CPS occur for parents and may operate on their health. It also brings much needed attention to a population whose trauma and health-harming experiences through this system are often neglected. Future research should more fully account for the system’s reach and impacts on parents – including attention to its effects on population health outcomes. To do this, improved data collection that includes linkable parent-level administrative data should be explored as part of a mandate for CPS to be more accountable to the populations that they intervene upon. The ubiquity of CPS interventions and mass separations of First Nations families found in our study, and the systemic racism, colonialism, poverty, and well-documented funding shortfalls by Canada’s government [50] that place First Nations families at greater risk of intervention also echo the clarion call by First Nations leaders for significant funding and resources to promote equity and wellness for First Nations families. This new investment should encompass significant funding to meet the basic needs of First Nations families through First Nations-led approaches to ensure guaranteed income, suitable housing, potable water, and community infrastructure, as well as resources for First Nations to re-assert jurisdiction over all matters relating to their children and families affirmed in 2019 by Canada’s Bill C-92 federal law (*An Act respecting First Nations*,* Inuit and Métis children*,* youth and families)*. This federal law aims to support development of Indigenous child welfare laws and services outside of colonial systems, including implementation of meaningful indicators for measuring outcomes that are based on a holistic vision of child well-being, such as those captured in the Measuring to Thrive Framework [51]. Investment is also required in First Nations-led preventative supports and the resurgence of holistic wellness for First Nations parents at risk of contact with CPS, including the establishment of community-based, supportive spaces outside of CPS where families in crisis can be referred as a first-line strategy to strengthen and keep families intact. For First Nations families who have experienced the harms of family separation, adequate funding for First Nations-led, culturally-based models that ensure long-term wellness for parents and the preservation of family and cultural bonds are needed, such as First Nations-led customary systems of care and reunification homes that allow parents and children to reside together with support. Policy interventions such as maintaining the full government social assistance/welfare benefit and public housing unit for the parent following a child being taken into temporary custody by CPS are also recommended so that parents are better supported to bring children home and prevent further family breakdown.

### Limitations

Our study findings should be interpreted within the context of several limitations. First, our analysis did not account for population-level rates of CPS contact at the intake/investigation level of the system, which are certain to be substantially higher than the rate of having an open file due to those that are reported being screened-out before or after the investigation stage. To provide a more accurate assessment of system reach, future research is needed that incorporate data on these first points of system contact. Second, due to most children in our data being only linkable to a single parent (the birthing parent), we were not able to reliably estimate rates of CPS contact among non-birthing parents. Using this approach, we are including birthing parents only in the denominator populations, which though mitigating duplication of coupled parents with only one birthing parents, may be double counting families with two or more birthing parents. Third, our study used the First Nations Research file to identify First Nations status, which is defined and controlled by Canada’s Federal Indian Act (1985), determining who is considered a “registered First Nations person”. Self-identified First Nations individuals and First Nations indviduals who never registered were included in the non-First Nations category, along with non-Indigenous birthing persons and Indigenous birth persons who were Métis or Inuit. Due to the importance identified by First Nations research partners of comparisons between First Nations and non-First Nations populations, these groups formed the basis for our analysis.

## Conclusions

Using whole-population data from one Canadian province, our study provides the first multiyear, population-level estimates of the prevalence of CPS contact among parents. We found exceptionally high rates of CPS involvement, with the burden falling hardest on First Nations parents, where 1 out of 2 were intervened upon by CPS. These data contribute to the quantitative literature on experiences of parents involved with North America’s CPS, a research area where there has been substantial neglect, and which - given it’s racialized and colonial dimensions - should constitute a key domain for health equity research. Our findings call for policies that intervene to support parents and mitigate the prevalent and potentially health-harming exposures they experience through this system, and highlight the need for future research that examines parental CPS contact across various populations, race/ethnicities, and jurisdictions. For First Nations parents, the striking level of CPS disruption and mass removals of children reinforce concerns about the contemporary system’s scope and the importance of considering its role in compounding health inequities and sustaining colonialism in Canada [3]. Findings encourage broad attention to the importance of First Nations-led models for the safeguarding, support and thriving of First Nations families outside and free of colonial systems.

## Electronic supplementary material

Below is the link to the electronic supplementary material.

Supplementary Material 1

## Data Availability

The datasets used for this study are not in the public domain, but researchers can access them by obtaining the necessary permissions and approvals.

## Abbreviations

CFSIS: Child and Family Services Information System
CI: Confidence interval
CPS: Child protective services
MCHP: Manitoba Centre for Health Policy
RD: Rate difference
RR: Rate ratio
TPR: Termination of parental rights
