# Supplementary material for: Inequities in child protective services contact among First Nations and non-First Nations parents in one Canadian province: a retrospective population-based study
Source: BMC Public Health. 2025 Apr 2;25:1224. doi: 10.1186/s12889-025-21813-5 (PMC11963421; doi:10.1186/s12889-025-21813-5)
Supplement: Supplementary file 1 — Supplementary Material 1 [file 12889_2025_21813_MOESM1_ESM.docx]

**SUPPLEMENTARY MATERIAL**

| **Supplementary Table 1**: Description of data sources. | | |
| --- | --- | --- |
| Name | Source | Description |
| First Nations Research File (MNRF) | First Nations Health and Social Secretariat of Manitoba (FNHSSM) | First Nations Research File: an extraction of all of the individuals in Manitoba who are registered under the Indian Act (Status) as of 2016. This file is under the ownership and control of the Manitoba First Nations through First Nations Health and Social Secretariat of Manitoba. Use of this file is contingent on approval from HIRGC. The file contains information such as a scrambled PHIN, sex, birth date, death date, residence code (used to identify on- vs off-reserve residence), and band affiliation. |
| Child and Family Services Information System (CFSIS) | Manitoba Families | All information on children in protective care and children and parents receiving services/monitoring from CPS comes from the Child and Family Services Information System. |
| Manitoba Health Insurance Registry (MHIR) | Manitoba Centre for Health Policy | The Manitoba Health Insurance Registry identifies the cohort of parents whose children were born in Manitoba, and identifies the biological parent-child relationships. The registry to generate area-level SES and income quintiles with linkages to the census. |
| Hospital Discharge Abstract (DAD) database | Manitoba Health | The DAD contains administrative, clinical (diagnoses and procedures/interventions), demographic, and administrative information for all admissions to acute care hospitals in Manitoba.Allows identifcation of all hospitalizations, including all birth hospitlizations. |

| **Supplementary Table 2**: Description of variables used. | | |
| --- | --- | --- |
| Variable | Source | Definition/Description |
| Ever had CPS file open for child | CFSIS | First event where CPS opened a file on a birthing parent any time at or after 9 months before the birth of their first child; or where a file was opened for any of the birthing person’s biological children after their birth; or where a CPS placement record was found for any of the birthing person’s biological children after their birth. |
| Ever had child(ren) in out-of-home placement | CFSIS | First event of CPS out-of-home placement of at least one biological child for a minimum of 7 days. An episode of placement is assessed based on placement start date and end date. |
| Termination of Parental Rights | CFSIS | First event of legal termination of parental rights based on a child’s legal status identfied in CFSIS as a ‘permanent ward’. |
| Age at first child's birth | Manitoba Health Insurance Plan Registry (MHIR), DAD | Recorded as the difference between the birthing person's birth date (idenfied in MHIR) and the date of birth of their first chlid (identified in hospital records, DAD). |
| Age at first parental contact with CPS | MHIR, CFSIS | Recorded as the difference between a birthing person's birth date (idenfied in MHIR) and the data of having a CPS file open at or after 9 months before the birth of their first child. |
| Rural/urban neighbour location | MHIR, Census | Rural areas include all towns and municipalities with an area population less than 10,000. |
| Neighbouhood income quintile | MHIR, Census | Dissemination area-level average household income values from the census are used to construct neighborhood income quintiles. The income quintile of the neighborhood is defined by methods developed at MCHP and are ranked from 1 (lowest income) to 5 (highest income); these quintiles are created separately for rural and urban Manitoba.94 Parents living in the lowest income neighborhoods (income quintile 1) are defined as living in neighborhood poverty, all other parents are defined as not living in neighborhood poverty. |
| First Nations status | FNRF | First Nations status identifcation based on individual being a registered First Nations person in FNRF. |
| On- vs off-reserve residence | FNRF | Place of residence of First Nations person identified as on vs. off reserve. |
